# Admission of tetanus patients to the ICU: a retrospective multicentre study

**DOI:** 10.1186/s13613-017-0333-y

**Published:** 2017-11-07

**Authors:** Rafael Mahieu, Thomas Reydel, Adel Maamar, Jean-Marc Tadié, Angeline Jamet, Arnaud W. Thille, Nicolas Chudeau, Julien Huntzinger, Steven Grangé, Gaetan Beduneau, Anne Courte, Stephane Ehrmann, Jérémie Lemarié, Sébastien Gibot, Michael Darmon, Christophe Guitton, Julia Champey, Carole Schwebel, Jean Dellamonica, Thibaut Wipf, Ferhat Meziani, Damien Du Cheyron, Achille Kouatchet, Nicolas Lerolle

**Affiliations:** 1 Département de réanimation médicale et médecine hyperbare, CHU Angers et faculté de santé Angers, 49933 Angers, France; 2 Service des Maladies Infectieuses et Réanimation Médicale, Maladies Infectieuses et Réanimation Médicale, CHU Rennes, 35033 Rennes, France; 3 Service de Réanimation Médicale, CHU de Poitiers, 2, rue de la Milétrie, 86021 Poitiers, France; 4 Département d’anesthésie-réanimation, LUNAM université, université d’Angers, CHU d’Angers, 49933 Angers, France; 5 Service de réanimation, Centre hospitalier Bretagne Atlantique, 56017 Vannes Cedex, France; 6 Medical Intensive Care Unit, Rouen University Hospital, Rouen, France; 7 Medical-surgical ICU, Hospital of Saint-Brieuc, 10 rue Marcel Proust, 22000 Saint-Brieuc, France; 8 Médecine Intensive Réanimation, Centre Hospitalier Régional et Universitaire de Tours, 37044 Tours, France; 9 Service de Réanimation Médicale, CHRU Nancy, Hôpital Central, Nancy, France; 10 Medical-Surgical ICU, Saint-Etienne University Hospital, Saint-Priest-en-Jarez, France; 11 Medical intensive care unit, Nantes academic hospital, Nantes university, Nantes, France; 12 Intensive Care Medicine, CHU de Grenoble, BP 218, 38043 Grenoble Cedex 9, France; 13 Service de Réanimation, Centre Hospitalier-Universitaire, Nice, France; 14 Service de Réanimation Médicale, Nouvel Hôpital Civil, Centre Hospitalo-Universitaire, Strasbourg, France; 15 Intensive Care Unit, University Hospital of Caen, Caen, France

**Keywords:** Tetanus, Intensive care unit, Outcome, Mechanical ventilation, Elderly patient, Prognosis, Ventilators, Mechanical, Aged, Comorbidity

## Abstract

**Background:**

An extended course of tetanus (up to 6 weeks) requiring ICU admission and protracted mechanical ventilation (MV) may have a significant impact on short- and long-term survival. The subject is noteworthy and deserves to be discussed.

**Methods:**

Twenty-two ICUs in France performed tetanus screenings on patients admitted between January 2000 and December 2014. Retrospective data were collected from hospital databases and through the registers of the town hall of the patients.

**Results:**

Seventy patients were included in 15 different ICUs. Sixty-three patients suffered from severe or very severe tetanus according to the Ablett classification. The median age was 80 years [interquartile range 73–84], and 86% of patients were women. Ninety per cent of patients (*n* = 63) required MV for a median of 36 days [26–46], and 66% required administration of a neuromuscular-blocking agent for 23 days [14–29]. A nosocomial infection occurred in 43 patients (61%). ICU and 1-year mortality rates were 14% (*n* = 10) and 16% (*n* = 11), respectively. Forty-five per cent of deaths occurred during the first week. Advanced age, a higher SAPS II, any infection, and the use of vasopressors were significantly associated with a lower number of days alive without ventilator support by day 90. Age was the only factor that significantly differed between deceased and survivors at 1 year (83 [81–85] vs. 79 [73–84] years, respectively; *p* = 0.03). Sixty-one per cent of survivors suffered no impairment to their functional status.

**Conclusion:**

In a high-income country, tetanus mainly occurs in healthy elderly women. Despite prolonged MV and extended ICU length of stay, we observed a low 1-year mortality rate and good long-term functional status.

## Background

Tetanus is caused by the neurological effects of the toxin produced by *Clostridium tetani.* Although it is a completely preventable disease, tetanus remains responsible for around 60,000 deaths per year worldwide [1]. The blockage of neuromuscular transmission by the toxin causes painful muscle spasms and respiratory distress requiring ICU admission and mechanical ventilation (MV) in about 80% of patients [2]. Considering the long-lasting effect of the toxin, prolonged ventilation combined with sedation and neuromuscular blockade up to 6 weeks may be required [2, 3]. In developing countries, where access to high intensity care may be a challenge, the mortality rate of tetanus has risen to 50% for a mean age of 50 years with little improvement over time [4, 5, 6, 7].

Data about severe tetanus are scarce in high-income countries. In Australia, a net decrease in tetanus-related mortality was observed between 1957 and 1985 (from 44 to 5%), which likely reflects the implementation of intensive care medicine over these years [8]. In developed countries, the enduring incidence of tetanus is mainly due to a lack of vaccination coverage of the elderly [9, 10]. In the USA and France, people over 65 years old have a twice to ten times greater risk of becoming infected with tetanus compared to younger patients [11, 12]. Elderly patients admitted to the ICU may be faced with a particularly high risk of poor outcome. Indeed, in elderly patients admitted to the ICU for medical reasons and requiring prolonged length of stay and/or MV, ICU- and 1-year mortality rates up to 50 and 70% have been reported, respectively [13, 14].

Treating tetanus in developed countries undoubtedly carries the challenge of prolonged ICU care for a particularly at-risk population. This may have a major impact on short- and long-term survival, which has not been described in recent years. We therefore conducted a multicentre retrospective study on such patients in France, reporting both short-term and 1-year mortality and long-term functional status.

## Patients and methods

### Study design

A retrospective cohort of adult tetanus patients was created. The study was conducted in 22 French ICUs. Patients were identified using hospital-based medical and administrative as well as ICU databases in each centre. All adult patients admitted to the ICU for tetanus from 1 January 2000 to 31 December 2014 were included.

### Data collection

Data on patient hospitalisations were retrieved from local ICU databases and medical files. Demographic data were collected, including age, sex, body mass index, Charlson Comorbidity Index, and Knaus’ classification of functional limitation (ratings are A for no limitation, B for slight functional limitation, C for severe functional limitation, and D for bed-ridden patients) [15]. Severity of acute illness was recorded according the Sequential Organ Failure Assessment (SOFA) [16], the Simplified Acute Physiology Score (SAPS) II [17], and the Ablett classification of tetanus severity (mild for mild trismus, no dysphagia, and no respiratory involvement; moderate for moderate trismus, dysphagia, and moderate respiratory involvement; severe for generalised spasticity, severe respiratory involvement; and very severe when associated with autonomic disturbance involving the cardiovascular system) [18]. Data on the clinical presentation of tetanus included the presence of a wound, incubation time, time from symptom to admission to the ICU, status of vaccination protection, severity and extent of spasms (isolated trismus, localised spasm outside the jaw, dysphagia, generalised tetanus), and presence of an autonomic dysfunction. Autonomic dysfunction was defined by the report of labile blood pressure or heart rate, or ventricular arrhythmia in medical files [19]. Length of stay in ICU, mortality in ICU, duration of MV, use of vasopressors, renal replacement therapy (RRT), and nosocomial infections were recorded. Durations of the administration of neuromuscular-blocking agents and sedatives were registered. The use of magnesium and verapamil was recorded. Surgical treatment of the wound, antimicrobial therapy, and the use and dosage of human tetanus immunoglobulin (HTIg) were recorded.

### Long-term outcome

Long-term survival outcome was obtained by consulting hospital databases and by consulting patient’s town hall registries. (The latter are used to record births and deaths, which is mandatory in France.) Last follow-up was determined depending on the date of inspecting town hall records and the day of admission into the ICU. Long-term functional status was defined using Knaus’ functional classification and the number of patients who required long-term care facilities. The functional status was retrieved from hospital medical records and general practitioners.

### Statistical analysis

We performed analyses using the SPSS v15.0 statistical software package (IBM, New York, USA). Continuous variables were reported as medians with 25–75% percentiles (IQR). Categorical variables were reported as n and percentage. All parameters were tested for 1-year survival and for the number of days alive without ventilator support by day 90. Continuous data were compared using Student’s *t* test or the Mann–Whitney test, as appropriate. Categorical variables were compared using Pearson’s Chi-squared test or Fisher’s exact test, as appropriate. Kaplan–Meier survival curves were used to evaluate mortality.

## Results

Fifteen ICUs identified 70 patients with tetanus over the study period. Baseline characteristics and clinical presentations of patients are provided in Table 1. Characteristics of tetanus are detailed in Table 2. Fifty-seven patients (81%) received antibiotic treatment (benzyl penicillin for 51 patients, metronidazole for 13, both antibiotics for 4, other regimen for 23) for a median duration of 7 days (IQR 7–10). Human tetanus immunoglobulin (HTIg) was used in the case of 57 patients (81%) after a median delay of 1 day after ICU admission, with a dose ranging from 250 to 5000 IU. None of the patients received verapamil to improve control of dysautonomia symptoms. Magnesium intravenous infusion was used in 23 patients for a median duration of 6 (IQR 2–14) days. Baclofen was used in 12 patients (17%), by intrathecal route in the case of 7 patients, for a median duration of 10 (IQR 4–12) days; all of them required MV. MV was performed in 63 patients (90%) for a median duration of 36 days (IQR 26–46; range 1–131). Continuous administration of a neuromuscular-blocking agent (NMBA) was used in 50 (71%) patients for a median duration of 23 days (IQR 14–29; range 1–39). Ninety-eight per cent (*n* = 61) of patients with severe or very severe tetanus were administered a continuous infusion of benzodiazepines for a median duration of 28 (IQR 19–34) days. Twenty-four patients (34%) required vasopressor support (only norepinephrine was used) for a median duration of 9 days (IQR 4–20; range 1–34). Two-thirds of patients underwent a tracheotomy (*n* = 46) after a median duration of 7 days (IQR 1–9; range 0–48) in the ICU. Median length of stay in the ICU and hospital was 41 days (IQR 24–53; range 1–117) and 51 days (IQR 32–69; range 1–178), respectively. Only one patient required RRT. Sixty-one per cent of patients (*n* = 43) developed a nosocomial infection with 12 bloodstream infections (4 central venous catheter-associated bloodstream infections, 2 cases of ventilator acquired pneumonia (VAP) with bacteraemia, 1 sinusitis, 2 pyelonephritis, 3 primitive blood stream infection), 32 (51% of ventilated patients) VAPs (including the two with associated bloodstream infection), and 1 *Clostridium difficile* infection. The VAP rate was 15 episodes per 1000 ventilator days.

**Table 1:** Baseline characteristics of patients

Parameters	Median [IQ] (min–max) or number (%)
Age	80 [73–84] (22–91)
Male sex	10 (14.3%)
Body mass index	24 [21–29]
Coexisting conditions	
Chronic heart failure	16 (23%)
Chronic respiratory failure	4 (6%)
Liver disease	0
Chronic kidney disease	4 (6%)
Diabetes	10 (14%)
Dementia	2 (3%)
Active cancer	2 (3%)
Charlson Comorbidity Index	4 [3–5]
Knaus’ classification	
Knaus A	35 (50%)
Knaus B	35 (50%)
Knaus C or D	0
SAPS II upon ICU admission	33 [26–41]
SOFA upon ICU admission	1 [0–3]

**Table 2:** Characteristics of tetanus and specific management

Parameters	Median [IQ] or number (%)
Status of protection	
No vaccination	26 (37%)
Vaccination > 10 years	18 (26%)
No information	26 (37%)
Wound	67 (96%)
Gardening wound	31 (44%)
Incubation time	10 [8–14]
Time from symptoms to admission	2 [1–3]
Ablett classification	
Mild	2 (3%)
Moderate	5 (7%)
Severe	29 (41%)
Very severe	34 (49%)
Trismus	70 (100%)
Localised spasm	34 (49%)
Dysphagia	46 (66%)
Generalised tetanus	39 (56%)
Autonomic dysfunction	40 (57%)
Blood pressure instability	29 (41%)
Heart rate instability	17 (24%)
Ventricular arrhythmia	4 (6%)

**Fig. 1:**
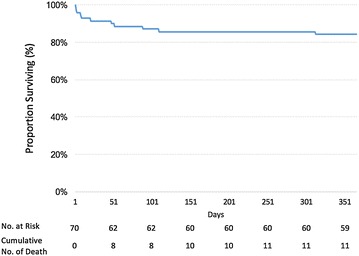
Kaplan–Meier curve for cumulative survival. Survival data are censored at one year. No patient was lost to follow-up at one year

ICU, 90-day, and 1-year mortality rates were 14% (*n* = 10), 13% (*n* = 9), and 16% (*n* = 11), respectively (see Fig. 1). The ICU mortality rate was higher than the 90-day survival rate due to one late death in the ICU of a patient at day 109. It is noteworthy that patients who did not receive MV had a mortality rate of 0% with a median length of stay in the ICU of 5 days (IQR 3–9; range 1–15), with all of them suffering from isolated cephalic tetanus. (Two patients had mild and five had moderate tetanus according to the Ablett classification.) Mortality status at 5 years was known for 57 patients (81%), among whom 36 (61% of the 57) were alive. Nearly half of the deaths that occurred during the first year occurred within the first week (*n* = 5). Four of these early deaths were caused by ventricular arrhythmia, possibly as a manifestation of autonomic dysfunction. Three patients died during the first 90 days in the ICU following the withdrawal of life-sustaining therapies, two patients died due to nosocomial infections, and one patient died after being discharged. None of the deaths could be attributed to delayed intubation resulting in hypoxic cardiac arrest. Among the survivors at last follow-up (*n* = 38), the health status according to Knaus’ classification was known for 95% of them, after a median duration of 1385 [302–3096] days. Sixty-one per cent of these survivors had no impairment of their functional status. Seventeen per cent (*n* = 6) of patients initially classified as Knaus A before ICU admission evolved to Knaus B (the same number was observed for Knaus B patients evolving to Knaus C) and 5% (*n* = 2) of Knaus A patients evolved to Knaus C. Only six patients (17% of survivors with a known functional status) required admission to a long-term care facility.

Age was the only factor that significantly differed between deceased and survivors at 1 year (83 [81–85] vs. 79 [73–84] years, respectively; *p* = 0.03). Advanced age, higher SAPS II (above the median of 33), any infection, bloodstream infection, VAP, and the use of vasopressors were significantly associated with a lower number of days alive without ventilator support by day 90 (see Table 3).

**Table 3:** Factors significantly associated with the number of days alive without ventilator support by day 90

Parameters	Number of days alive without ventilator support by day 90. Median [interquartile range]	*p* value
Age (years)		
≥ 80	43 [0–57]	0.03
< 80	53 [45–59]	
SAPS II^a^		
≥ 34	44 [22–54]	0.04
< 34	57 [43–63]	
Any infection		
Yes	45 [27–57]	0.01
No	60 [48–71]	
Bloodstream infection		
Yes	42 [6–51]	0.04
No	54 [36–52]	
Ventilator acquired pneumonia		
Yes	42 [19–57]	0.01
No	55 [47–63]	
Vasopressor use		
Yes	43 [0–57]	< 0.05
No	53 [42–62]	

^a^Parameter dichotomised at median value

## Discussion

### Epidemiology of tetanus in developed countries

In this study, tetanus mainly occurred in elderly patients (75% of patients were older than 73, median age 80 years), which is consistent with previous Italian and French studies reporting a median age of 76 years or 86% of patients being 70 years old or over, respectively [12, 20]. This is in contrast with US reports, which involved younger patients (median age of 49 years) [11]. The description of tetanus in injection drug users in the US [11] may explain part of these differences given that in our study a gardening wound was the main portal of entry in our patients. Women are a particular at-risk population (86% of tetanus patients in this study and 68% in Italy). It may be hypothesised that mandatory vaccination during compulsory military service for men provides them with long-lasting immunisation [20].

### Controlling muscle spasms and autonomic dysfunction

Several treatments have been tested to control autonomic instability and muscle spasm and to hasten recovery. Despite little evidence, benzodiazepines remain the main treatment regimen for tetanus spasms [21]. Benzodiazepines in continuous infusion were used in all severe or very severe tetanus patients except one, while NMBAs were required in the majority of cases. Baclofen was used only marginally. Baclofen has the potential to relieve muscle spasms and may reduce the need for ventilation; however, evidence in favour of baclofen use is limited to case studies with conflicting results [22]. Severe autonomic dysfunction, and in particular early-onset ventricular arrhythmia, was responsible for 36% of deaths is this study; this percentage is commonly reported in tetanus studies [4, 23, 24]. The pathophysiological link between ventricular arrhythmia and tetanus toxin is still unclear. Verapamil and magnesium have been suggested as a prevention method. None of our patients received verapamil, while one-third of patients received magnesium treatment. We did not observe any association between magnesium use and outcome; however, this study was not designed to draw conclusions on this hypothesis. As shown by Thwaites et al. [2], magnesium may reduce autonomic dysfunction thanks to its calcium-antagonist properties [25]; however, no benefits for mortality were observed in our study, which is consistent with a recent meta-analysis [25].

### Neutralisation of toxin, antibiotic, and wound management

HTIg was used in more than 80% of patients, with no difference in outcome between the patients who received HTIg treatment and those who did not (data not shown). HTIg is conventionally recommended in tetanus to bind the unbound toxin in serum, which has been demonstrated in 10% of serum samples [26]. Notably, none of the patients in this study received HTIg by intrathecal route, while previous uncontrolled studies showed an association between this route of administration and reduced mortality [27]. More recently, the benefits of intrathecal route combined with intramuscular HTIg were assessed in a randomised study and only a reduction in hospital stay was observed [28]. Antimicrobial therapy probably plays a minor role in tetanus but is conventionally recommended to halt the toxin’s production [21, 29]. No difference was observed in this study between patients treated with penicillin, metronidazole, or other regimens (data not shown). The first study that compared penicillin to metronidazole found a greater reduction in mortality in the metronidazole group [30]; however, more recent studies, including a randomised study, did not show any difference between these treatments [4, 31]. Penicillin and metronidazole are therefore equally recommended. Wound debridement, which can eradicate persistent spores of *C. tetani*, was only performed in 16% of cases. It is likely that in a context of a rare disease with severe symptoms, the portal of entry (sometimes a very small one) did not appear as a priority. However, persistence of *C. tetani* in the wound has been described despite antimicrobial therapy, and wound debridement therefore seems essential to haste the eradication of the bacteria [32].

### Prolonged mechanical ventilation and elderly patients

Most patients in our study required prolonged mechanical ventilation. Most of them were elderly (median age 80 years), but all had no or only slight functional limitation and a low burden of comorbidities as measured by Knaus’ classification and the Charlson Comorbidity Index. Indeed, a gardening wound was identified in about half of the cases, thereby selecting a population of “healthy elderly”. Despite a strikingly high duration of MV with frequent requirement for tracheotomy and a long ICU length of stay, ICU and 1-year survival were excellent, at 86 and 84%, respectively. The increasing number of elderly patients in the ICU, combined with concerns regarding their high mortality rate and uncertainty regarding the functional outcome, fuelled a continuous debate about the benefits of their admission [13, 33–38]. A recent study by Moitra showed that higher duration of MV and length of stay in the ICU were almost linearly correlated with outcome [13]. In sharp contrast with this late study, the very low rate of comorbidities in our population combined with a completely reversible disease may explain the very low ICU and 1-year mortality rate in our study [13, 14]. Finally, our study confirms and builds on a study performed in Italy that showed a 16.5% hospital mortality rate for tetanus patients in a population with a median age of 76 years [20]. Further comparison with this study is limited by the lack of any ICU data and a known outcome for only 43.9% of patients.

### Outcome of tetanus in high-income countries

The low mortality rate observed in our patients (16% 1-year mortality) in comparison with low-income countries likely reflects the availability of high-cost ICU facilities [8]. These are essential for managing prolonged MV, paralysing agents, autonomic dysfunction, and the high rate of infectious complications. Indeed, the mortality rate of severe tetanus patients remains between 30 and 50% in low-income countries [4, 5, 6], which is consistent with the mortality rate of tetanus before the implementation of ICUs [39] in Europe. A study in the 1970s reported a mortality rate of only 11% in England [40].

## Limitations

Our study presents certain limitations. The main concern is the lack of detailed information regarding the long-term functional status of survivors. Indeed, we could not assess it precisely due to the study’s design. However, the functionality score according to Knaus’ classification was known for 95% of survivors, with 61% of them suffering no loss of autonomy. Moreover, only six patients required admission to a long-term care facility. Another limitation is that we could not include tetanus patients subject to a Do-Not-Resuscitate order that would have prevented ICU admission, or patients who suffered hypoxic cardiac arrest before ICU admission. Finally, due to the incompleteness of most medical reports, we could not establish accurately the processes or indications that lead the physicians to intubate, start NMBA treatment or perform a tracheotomy.

## Conclusion

Tetanus in France occurs mainly in healthy elderly patients, especially women. In this population, despite prolonged MV with frequent NMBA use as well as extended ICU length of stay, we observed a low mortality rate and a good long-term functional status.

## Abbreviations

HTIg: human tetanus immunoglobulin
ICU: intensive care unit
MV: mechanical ventilation
NMBA: neuromuscular-blocking agent
RRT: renal replacement therapy
SAPS II: Simplified Acute Physiology Score II
SOFA: Sequential Organ Failure Assessment
VAP: ventilator-associated pneumonia
